# Cardiogenic Shock Due to Unsuspected Tachycardia in a Child

**DOI:** 10.1016/j.jaccas.2022.05.025

**Published:** 2022-07-20

**Authors:** Kosuke Nishikawa, Yo Kajiyama, Hirokazu Shiraishi, Keitaro Senoo, Masaaki Yamagishi

**Affiliations:** a Department of Pediatrics, Kyoto Prefectural University of Medicine, Kyoto, Japan; b Department of Cardiovascular Medicine, Kyoto Prefectural University of Medicine, Kyoto, Japan; c Department of Cardiac Arrhythmia Research and Innovation, Kyoto Prefectural University of Medicine, Kyoto, Japan; d Division of Pediatric Cardiovascular and Cardiovascular Surgery, Department of Surgery, Kyoto Prefectural University of Medicine, Kyoto, Japan

**Keywords:** ablation, acute heart failure, appendectomy, complication, pediatric surgery, tamponade, AT, atrial tachycardia, ECG, electrocardiogram, ECMO, extracorporeal membrane oxygenation, LAA, left atrial appendage, LV, left ventricle, RFCA, radiofrequency catheter ablation

## Abstract

A 10-year-old girl experienced cardiac failure due to atrial tachycardia originating from a left atrial appendage. Surgical appendectomy was done after a recurrence of the atrial tachycardia just after the first attempt at catheter ablation. A second ablation attempt was avoided because of the risk of cardiac perforation. (**Level of Difficulty: Intermediate.**)

## Central Illustration

**Figure:**
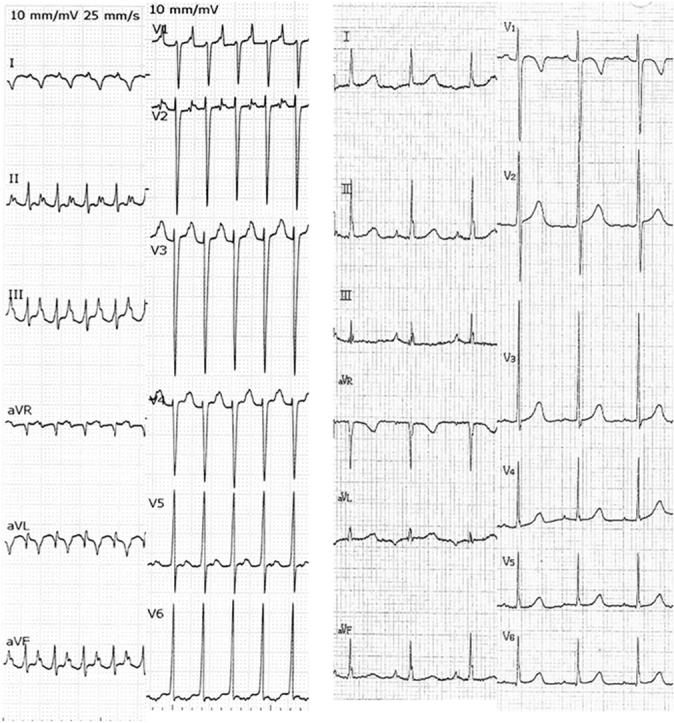

A 10-year-old, previously healthy girl was admitted to a hospital after experiencing general fatigue for a month. She had tachycardia, hypotension, cardiomegaly, and pulmonary congestion. She was referred to our hospital for intensive treatment because of acute heart failure.

**Learning Objectives**

• To understand the risk factors of cardiac perforation in catheter ablation.
• To know the management strategies, including catheter techniques and surgical treatments for arrhythmogenic substrates and complications due to treatments.

When she was admitted to our hospital, she was conscious and afebrile, even though pale and with cold extremities. Her heart sounds had no significant murmur but an irregular rhythm. Her average heart rate was 190 beats/minute, irregularly varying from 180 to 200 beats/min. Her blood pressure was 94/57 mm Hg, supported by continuous catecholamine infusion (dopamine 5 μg/kg/min, dobutamine 5 μg/kg/min). Her respiratory sounds had coarse crackles without hypoxia.

## Medical History

Her medical history was insignificant. She had no family history of cardiac disease.

## Differential Diagnosis

Possible diagnoses included acute or subacute myocarditis, metabolic cardiomyopathy, idiopathic cardiomyopathy, congenital heart disease, and tachycardia-induced cardiomyopathy.

## Investigations

**Figure 1:**
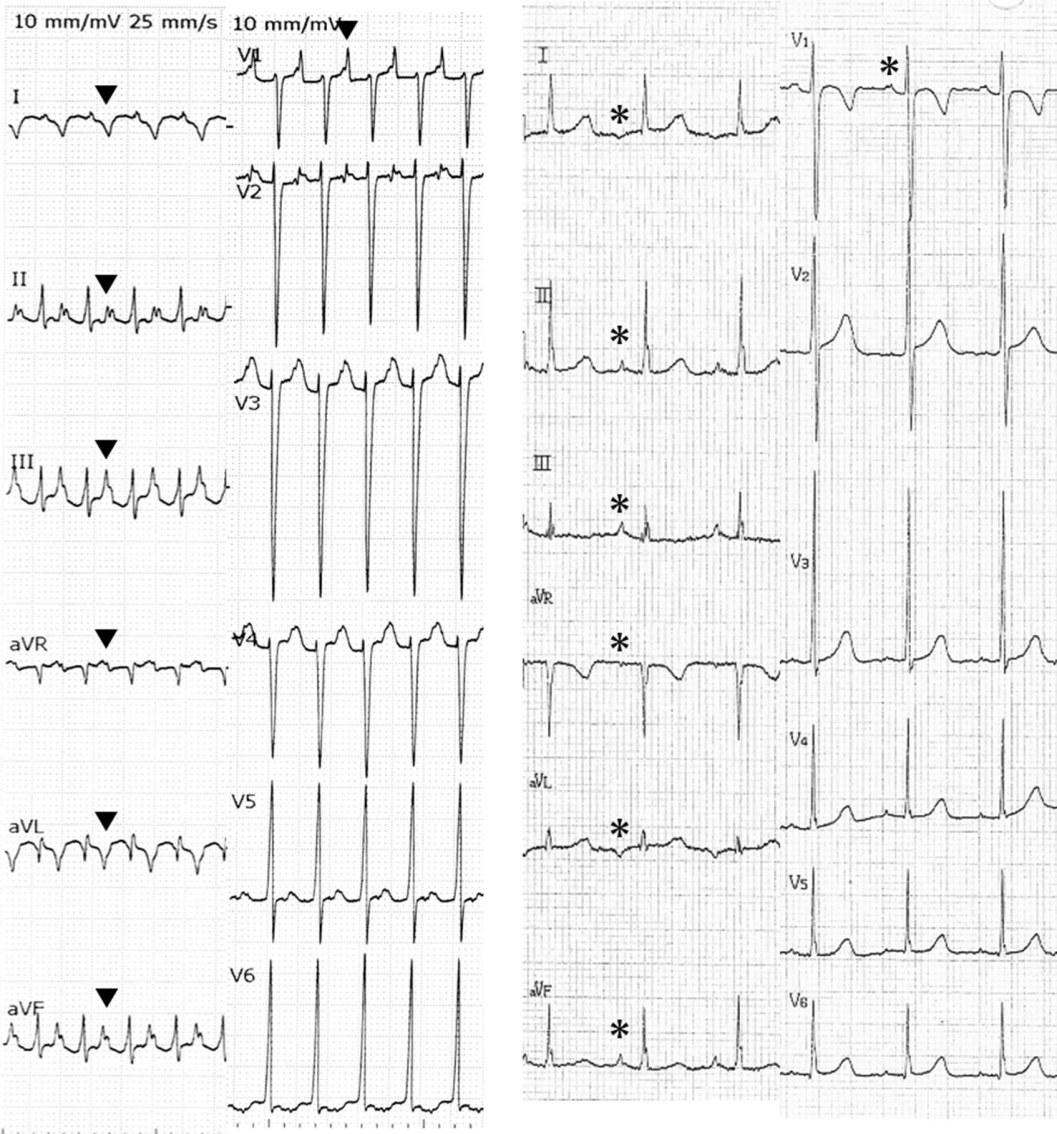
Electrocardiograms **(Left)** On admission. **(Right)** When patient was 6 years old. P-wave on admission **(arrowhead)** and 4 years ago **(asterisk)**.

Laboratory results included normal values of white blood cell count, electrolytes, and C-reactive protein. The serum levels of liver enzymes (aspartate transaminase and alanine aminotransferase) were mildly elevated. The brain natriuretic peptide and creatine kinase were elevated significantly ≤1,569.3 pg/mL and 408 U/L. An electrocardiogram (ECG) revealed an incessant atrial tachycardia (AT) (atrial rate 210 beats/min), that is, a negative P-wave in leads Ⅰ and aVL, and a positive P-wave in leads Ⅱ, Ⅲ, aVF, and V_1_ (Figure 1). The Wenckebach phenomenon decreased the ventricular rate to 150 to 180 beats/min. Cardiomegaly and pulmonary congestion were observed on a chest x-ray. Echocardiography indicated severe left ventricular (LV) systolic dysfunction with an impaired ejection fraction of 0.28, an enlarged internal diastolic dimension of 52.0 mm (Z value = 4.5), and moderate mitral valve regurgitation without any other structural abnormality.

We reviewed the ECG performed when she was 6 years old during a school medical examination and concluded that the AT rhythm was observed but overlooked (Figure 1). The AT rhythm might have continued and accelerated for 4 years.

## Management

A provisional diagnosis of tachycardia-induced cardiomyopathy was made, and antiarrhythmic therapy was started. After the initiation of sedative drugs, she manifested severe hypotension and extracorporeal membrane oxygenation (ECMO) was used. After that, the AT rate varied from 140 beats/min in a sleepy state to 180 beats/min with stimuli, although the atrial activation pattern did not change in ECG. Continuous infusions of amiodarone and landiolol hydrochloride were administered for rate control but were not successful.

**Figure 2:**
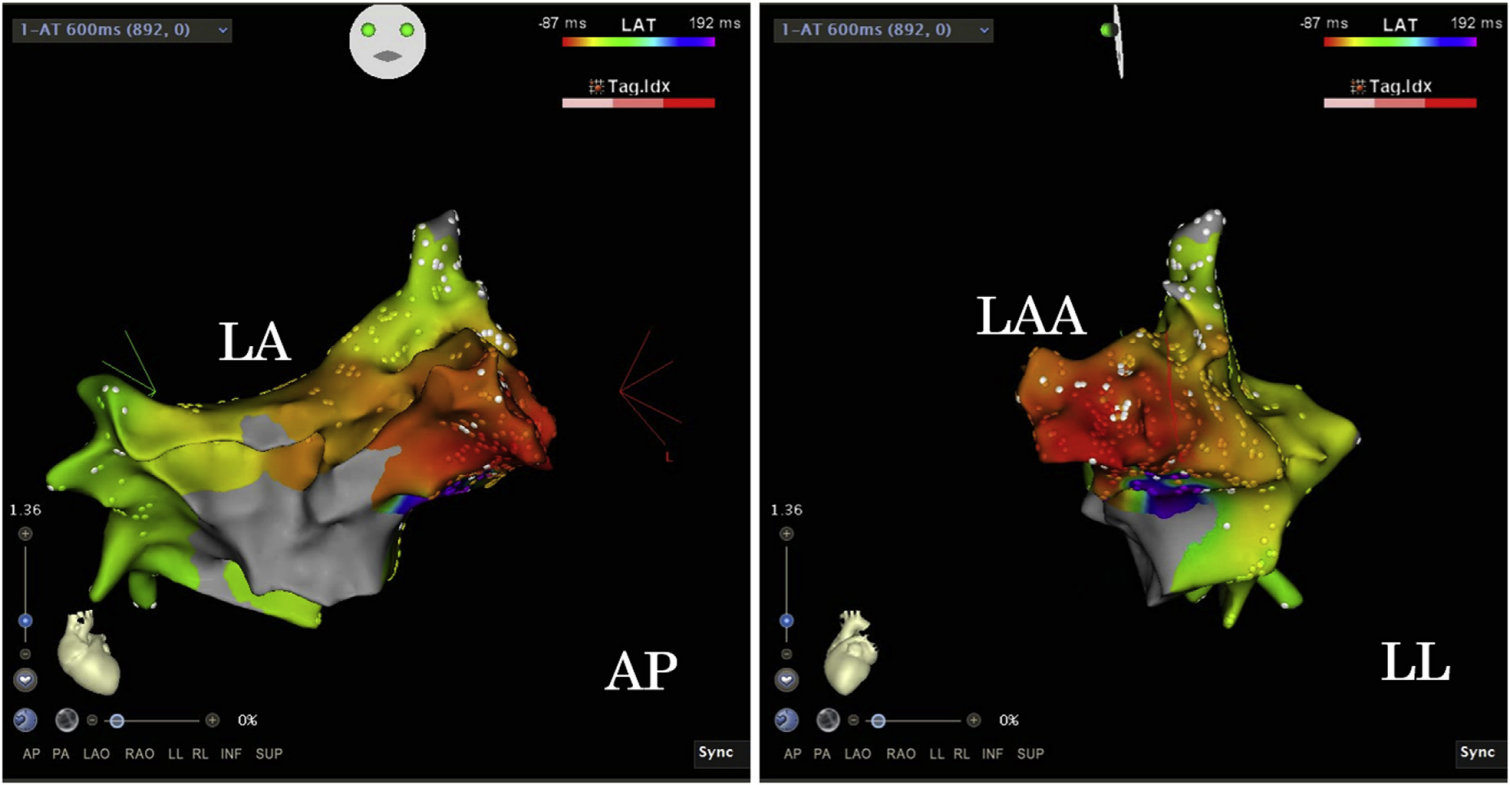
Electroanatomical Activation Map AP = anteroposterior view; LA = left atrium; LAA = left atrial appendage; LL = left lateral view.

**Figure 3:**
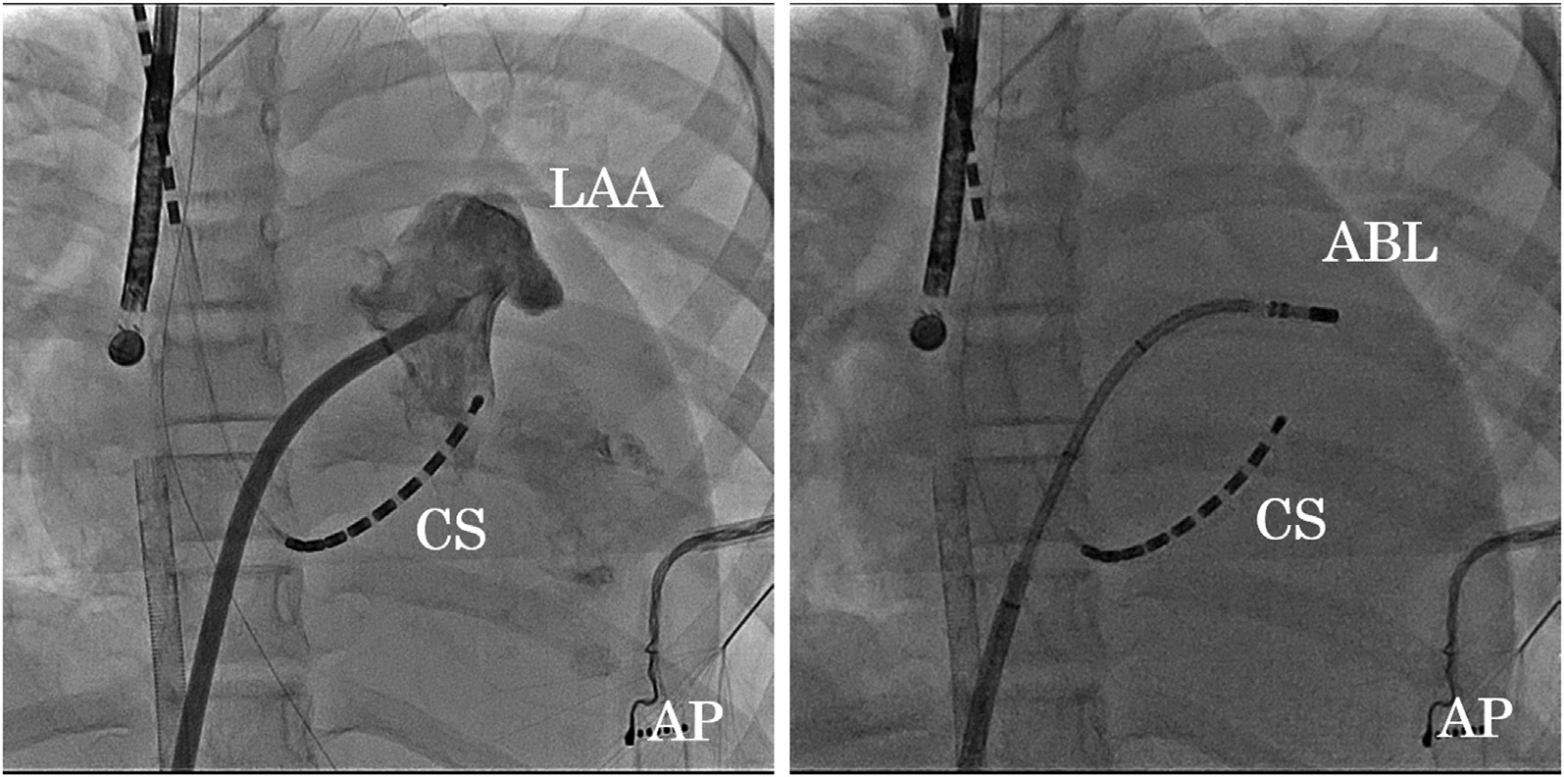
Successful Ablation Site of LAA **(Left)** Cardiac angiography of the LAA. **(Right)** AT stopped after the catheter ablation. ABL = ablation catheter; AP = anteroposterior radiographic view; AT = atrial tachycardia; CS = coronary sinus; LAA = left atrial appendage.

Therefore, radiofrequency catheter ablation (RFCA) was performed 7 days after admission under ECMO support. An electrical activation map with use of the Carto system (Biosense Webster) through a transseptal puncture revealed a focal origin of the AT in the left atrial appendage (LAA) (Figure 2). An irrigated bidirectional ThermoCool SMARTTOUCH (Biosense Webster) ablation catheter was placed and confirmed the earliest A wave at the base of the LA. The RFCA was performed with a power output of 30 W at that point, then the AT stopped after the 11th attempt at power delivery. The success point was the anteroinferior of the base of the LAA, but contact force of the ablation catheter might have been insufficient to avoid LA perforation even with use of a steerable sheath (Figure 3). Three additional RF deliveries were given at the success point of the AT. However, recurrence of the AT was observed 3 hours after the intervention, so she was kept under ECMO support because of the tachycardia.

A surgical LAA resection was performed the next day, and the AT ceased immediately just after the appendectomy. A weaning process of ECMO was started on the next day after the surgery, and ECMO was discontinued on the fifth day after the operation; catecholamine support was continued. Her LV systolic function gradually improved. She did not present any arrhythmia after the operation and was discharged on the 55th day without any sequelae.

**Figure 4:**
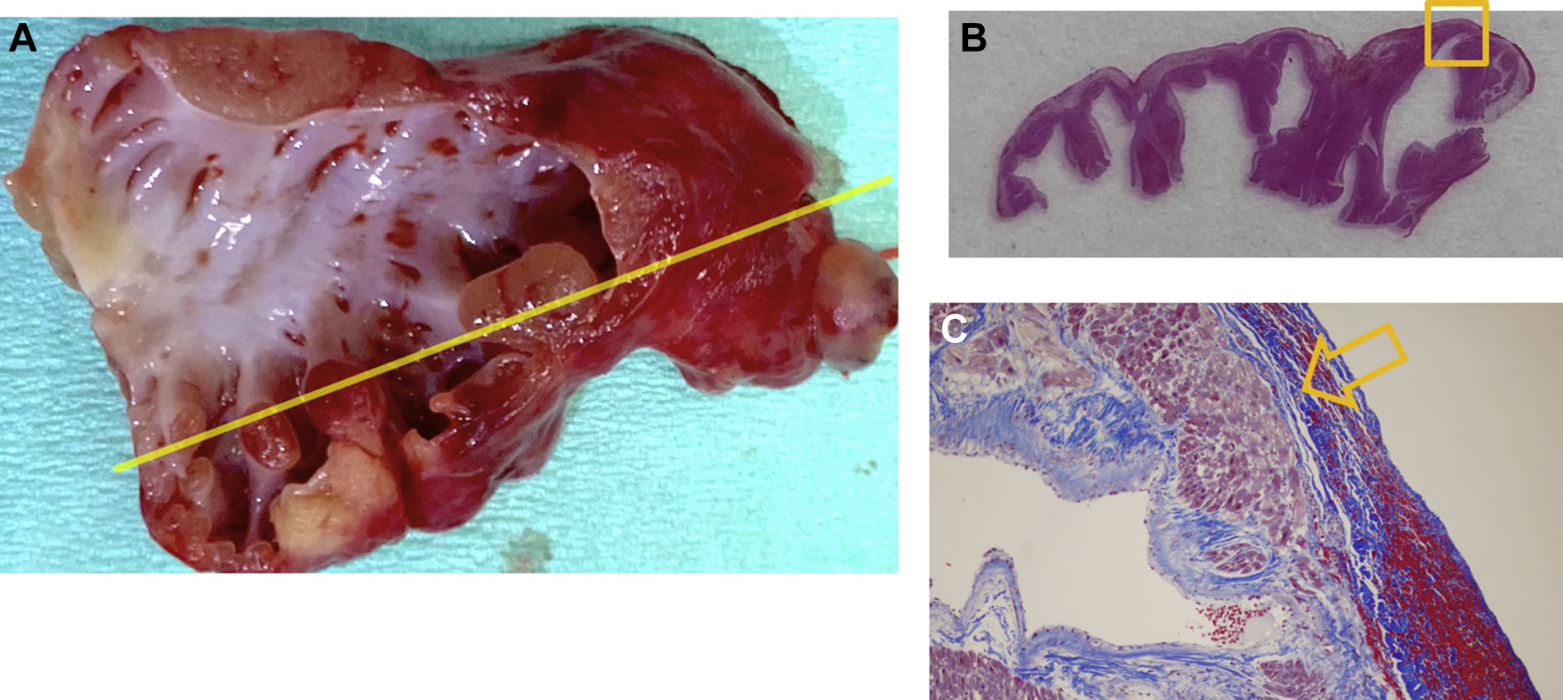
Gross and Histologic Appearance **(A)** Gross appearance: no signs of perforation were observed. Tissue slicing was done on the **yellow line**. **(B)** Tissue slicing revealed coarse trabeculations of pectinate muscles. The **yellow box** indicates the area magnified in **C**. **(C)** Masson’s trichrome stained section, ×100 magnification. The histologic change reached just beneath the epicardium **(yellow arrow)**.

Pathologic study of the resected tissue was performed. Histologic staining with Masson’s trichrome method revealed that transmural lesions made by the RF ablation, such as edema, coagulation, and loss of striations, reached just beneath the epicardium of the LAA (Figure 4).

## Discussion

The RFCA can result in some degree of unexpected adverse events, and the LAA is one of the risky areas for cardiac perforation during the RFCA because of its thinner wall. A recent study reported that the incidence of cardiac perforation during catheter ablation for atrial fibrillation had decreased to 0.61%, and absence of intracardiac echocardiography increased the risk of cardiac complications.^1^ Di Biase et al^2^ reported that successful treatment by use of a distal tip of the LAA needs to be instrumented and with careful technique.

In our case, the P-wave morphology was typical of the LAA origin.^3^ The patient was under circulatory support by ECMO through the left femoral vessels, and an RFCA catheter was inserted through the right femoral vein. We used procedural monitoring with transesophageal echocardiography to avoid vessel injury by an additional sheath insertion for intracardiac echocardiography in the young patient. This supportive device enabled us to conduct the RF needle for atrial septum puncture and to monitor cardiac situations throughout the session.

Several case reports on epicardial ablation for the LAA highlighted the difficulty of endocardial ablation because of reticulated pectinate muscles and variations in tissue thickness.^4^^,^^5^ Guo et al^6^ observed that approximately 28% of patients with focal AT originating from the right or left atrial appendage were finally treated by appendectomy after RFCA failure.

Our case resulted in a transient success of cessation, although recurrence was observed after the session. We decided on the surgical approach instead of retrying the RFCA because early weaning from ECMO was recommended for avoiding ECMO-related complications.

Our case is instructive in the sense that it revealed the depth and severity of histologic changes of the LAA made by the RFCA in a pediatric patient examined the next day after the procedure. Pathologic assessment of the ablation lesion showed the appearance of a distinct zone of coagulative necrosis with edema just beneath the epicardium. The risk of cardiac perforation by a second RFCA attempt might increase as the result of a fragile lesion because remodeling with fibroplasia and fibrosis would increase beyond 7 days after the ablation procedure,^7^ and cardiac perforation would be critical during anticoagulation therapy in support of ECMO.

## Follow-Up

One year after surgical treatment, the patient maintained normal sinus rhythm, and the LV function recovered fully with an ejection fraction of 0.62 and normal LV volume.

## Conclusions

AT originating from the LAA was successfully treated with RFCA and surgery. Because of the thin wall of the LAA and the high risk of cardiac perforation, this case report underlines the importance of choosing appropriate approaches depending on the peculiarities of individual patients and consideration of the risks and benefits.

## Funding Support and Author Disclosures

The authors have reported that they have no relationships relevant to the contents of this paper to disclose.
